# Functional Characterization of *Verticillium dahliae* Race 3-Specific Gene *VdR3e* in Virulence and Elicitation of Plant Immune Responses

**DOI:** 10.1128/spectrum.01083-23

**Published:** 2023-06-28

**Authors:** Qian Tan, Ran Li, Lei Liu, Dan Wang, Xiao-Feng Dai, Li-Min Song, Dan-Dan Zhang, Zhi-Qiang Kong, Steve J. Klosterman, Toshiyuki Usami, Krishna V. Subbarao, Wen-Xing Liang, Jie-Yin Chen

**Affiliations:** a Shandong Engineering Research Center for Environment-Friendly Agricultural Pest Management, College of Plant Health and Medicine, Qingdao Agricultural University, Qingdao, People’s Republic of China; b State Key Laboratory for Biology of Plant Diseases and Insect Pests, Institute of Plant Protection, Chinese Academy of Agricultural Sciences, Beijing, People’s Republic of China; c Western Agricultural Research Center, Chinese Academy of Agricultural Sciences, Changji, People’s Republic of China; d Institute of Vegetables and Flowers, Chinese Academy of Agricultural Sciences, Beijing, People’s Republic of China; e United States Department of Agriculture, Agricultural Research Service, Salinas, California, USA; f Graduate School of Horticulture, Chiba University, Matsudo City, Japan; g Department of Plant Pathology, University of California—Davis, c/o U.S. Agricultural Research Station, Salinas, California, USA; Beijing Forestry University

**Keywords:** *Verticillium dahliae*, effector, immunity, race, pathogen-associated molecular pattern, PAMPs, virulence

## Abstract

*Verticillium dahliae* is a soilborne fungal pathogen that causes disease on many economically important crops. Based on the resistance or susceptibility of differential cultivars in tomato, isolates of *V. dahliae* are divided into three races. Avirulence (*avr*) genes within the genomes of the three races have also been identified. However, the functional role of the *avr* gene in race 3 isolates of *V. dahliae* has not been characterized. In this study, bioinformatics analysis showed that VdR3e, a cysteine-rich secreted protein encoded by the gene characterizing race 3 in *V. dahliae*, was likely obtained by horizontal gene transfer from the fungal genus *Bipolaris*. We demonstrate that VdR3e causes cell death by triggering multiple defense responses. In addition, VdR3e localized at the periphery of the plant cell and triggered immunity depending on its subcellular localization and the cell membrane receptor BAK1. Furthermore, VdR3e is a virulence factor and shows differential pathogenicity in race 3-resistant and -susceptible hosts. These results suggest that VdR3e is a virulence factor that can also interact with BAK1 as a pathogen-associated molecular pattern (PAMP) to trigger immune responses.

**IMPORTANCE** Based on the gene-for-gene model, research on the function of avirulence genes and resistance genes has had an unparalleled impact on breeding for resistance in most crops against individual pathogens. The soilborne fungal pathogen, Verticillium dahliae, is a major pathogen on many economically important crops. Currently, *avr* genes of the three races in V. dahliae have been identified, but the function of *avr* gene representing race 3 has not been described. We investigated the characteristics of VdR3e-mediated immunity and demonstrated that VdR3e acts as a PAMP to activate a variety of plant defense responses and induce plant cell death. We also demonstrated that the role of VdR3e in pathogenicity was host dependent. This is the first study to describe the immune and virulence functions of the *avr* gene from race 3 in V. dahliae, and we provide support for the identification of genes mediating resistance against race 3.

## INTRODUCTION

Verticillium wilt is a soilborne fungal disease caused by Verticillium dahliae (1). V. dahliae can infect more than 660 plant species from 38 families, including more than 180 crops, such as cotton, lettuce, potato, strawberry, sunflower, and tomato (2, 3). The infection process of V. dahliae begins with the germination of the residual microsclerotia or conidia in the soil in response to root exudates (4). Hyphae invade roots and grow through the cells toward the vascular tissues (5, 6). Large numbers of conidia and microsclerotia are produced in the xylem, both clogging the vessels and secreting proteins that modulate host defenses and hormone levels, leading to wilting or defoliation of the plants (7, 8). Because the microsclerotia of V. dahliae are long-lived, Verticillium wilt is especially difficult to control (9, 10).

Resistance in tomato to Verticillium wilt was first described in 1951 and depends on a locus called *Ve* (11), which confers resistance to race 1 of V. dahliae (12). Cultivars carrying the *Ve* locus were deployed in tomato production for years, although resistance-breaking race 2 strains had appeared in tomato fields and supplanted race 1 strains (13). Subsequently, V. dahliae was divided into two additional races with the development of tomato rootstock cultivars that were resistant to race 2. A single dominant locus, V2, controlling the resistance to race 2 strains was identified (14). Isolates previously described as race 2 that could overcome this resistance were then described as race 3 (14). Although Verticillium wilt resistance is described in many crops (15–17), the designation of races was only possible in tomato (18) and lettuce (19, 20), owing to the availability of differential cultivars in these crops.

The *Ve* locus contains two homologous genes, *Ve1* and *Ve2*, both of which encode a cell surface receptor in the extracellular leucine-rich repeat (LRR) receptor-like protein family (12). Subsequently, only *Ve1* was demonstrated to mediate this resistance in tomato against the race 1 strains of V. dahliae (21). Recent studies have demonstrated that Ve1 and Ve2 can combine to form heteromeric complexes to enhance specific immunity to the race 1 strains of V. dahliae through a rapid burst of reactive oxygen species (22). After the transfer of *Ve1* into Arabidopsis thaliana, *Ve1* remained fully functional and imparted resistance to race 1 strains of V. dahliae (23). Transgenic tobacco and cotton that expressed *Ve1* constitutionally showed resistance to race 1 (24). The cytoplasmic tail and LRR regions of Ve1 are essential for its function and interact with the receptor-like kinase suppressor of BIR1-1 (SOBIR1) via its C terminus (25, 26). *Ve1* is differentially induced in resistant and susceptible plants, and its transcription level is indirectly regulated by defense/stress hormones (27). Homologs of functional Ve1 are conserved in plant species inside and outside the *Solanaceae* family (28). Based on the genome sequencing and comparative genome analysis of race 1 and 2 strains of V. dahliae, it was confirmed that *Ave1* acts as an *avr* gene in race 1 strains and is recognized by the resistance gene *Ve1* in tomato to induce immune responses (29). Recognition of the effector protein Ave1 of V. dahliae by immune receptor Ve1 triggers the hypersensitive response (HR) in tomato and tobacco but does not induce HRs in A. thaliana (30, 31). Ave1 can also induce the expression of defense genes independently of Ve1 (32).

The signaling pathway that Ve1 relies on for the recognition of Ave1, partially overlaps in tomato and A. thaliana (23). Normally, enhanced disease susceptibility 1 (EDS1) is involved in TIR-NB-LRR-mediated signaling, and non-race-specific disease resistance 1 (NDR1) is involved in CC-NB-LRR-mediated signaling (33). However, both EDS1 and NDR1 are required for *Ve1*-mediated resistance (21). In addition to this, the signaling pathway that modulates Ve- and Ave1-triggered immune responses also requires the participation of Mek2, which is a mitogen-activated protein (MAP) kinase gene (21). Mek2 is also involved in *Pto*-mediated resistance against Pseudomonas syringae (34). Nrc1, an NB-LRR protein, is necessary for multiple resistance proteins to induce an HR (35) and is also indispensable in the immune signaling pathways triggered by Ve1 and Ave1 (21). The F-box protein Acif1, which plays a role in Cf4/9-mediated resistance against *Cladosporium fulvum*, also plays an important role in the Ve1-mediated resistance pathway (21, 36). BRI1-associated kinase-1 (BAK1) is required for LLR-receptor-like proteins (LRR-RLPs) and/or LRR-receptor-like kinase (LRR-RLKs). In contrast, SOBIR1 is required for LRR-RLP function. These kinases have been shown to play important roles in immune receptors triggering defense responses (37). BAK1 is also required in the Ve1-mediated resistance pathways in response to V. dahliae (21).

Further comparative genomic and biological analyses led to the discovery of *avr* genes from V. dahliae race 2 and 3 strains. Av2 was identified as an effector by comparative population genomics analysis of race 1, 2, and 3 strains and its interactions with tomato plants carrying *V2* (38). Yet another study systematically analyzed the lineage-specific regions in different races of V. dahliae by comparative and functional genomics, revealing the secreted protein VdR3e that contributes to the virulence of race 3 strains (39). Thus, avirulence genes have been identified in each of the three V. dahliae races, but functional characterization of VdR3e has been lacking. In previous experiments, VdR3e induced host cell death, suggesting that VdR3e can induce immune responses similar to effectors secreted by races 1 and 2. The purpose of this study was to elucidate the role of VdR3e in virulence.

## RESULTS

### Bioinformatics analysis of VdR3e from *Verticillium dahliae*.

**FIG 1:**
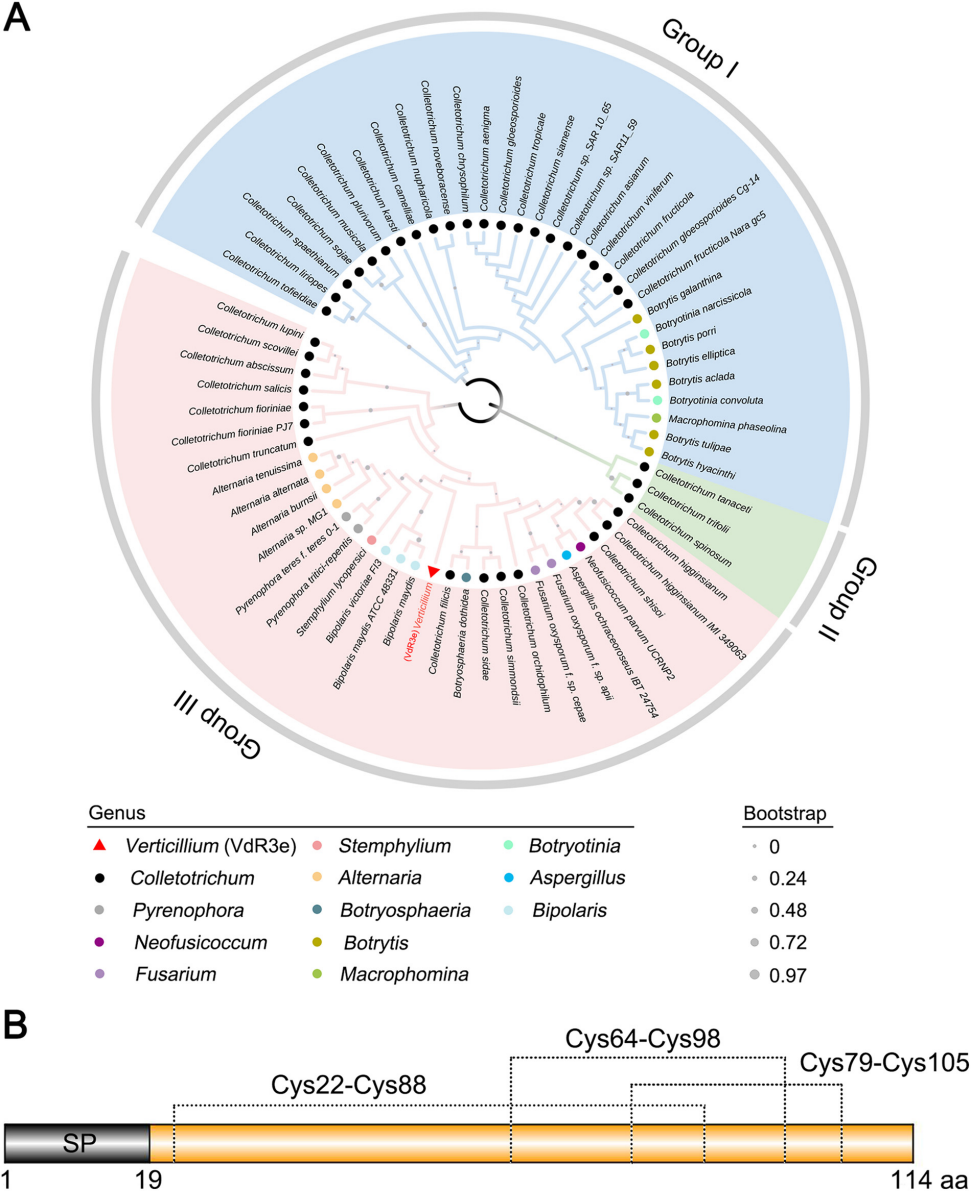
Results from the bioinformatics analysis of VdR3e from V. dahliae. (A) Construction of phylogenetic tree of VdR3e. The phylogeny was constructed using MEGA 5.05, with maximum likelihood (parameters: 1,000 bootstraps, Jones-Taylor-Thornton model). The position of VdR3e is marked with a red triangle. Homologous proteins of different genera were marked by dots of different colors (as shown in the legend). (B) Structural prediction of VdR3e. The signal peptide of VdR3e was predicted using an online signal peptide prediction tool SignalP 5.0. The disulfide bond of VdR3e was predicted based on online tool DiANNA.

To investigate the evolution of VdR3e, BLAST searches were performed with the VdR3e sequence using the NCBI database. Among the protein sequences obtained from BLAST search, those whose sequence identity with VdR3e was >30% and had a protein length of <320 amino acids were considered as VdR3e homologs. Thus, VdR3e homologous proteins were present in only 12 fungal genera (*Colletotrichum*, *Pyrenophora*, *Neofusicoccum*, Fusarium, *Stemphylium*, *Alternaria*, *Botryosphaeria*, *Botrytis*, *Macrophomina*, *Botryotinia*, Aspergillus, and *Bipolaris*), among which VdR3e shared the highest homology with *Colletotrichum* proteins, and most of these were uncharacterized. A phylogenetic tree containing VdR3e and homologous proteins from 63 species of fungi was constructed. Phylogenetic analysis showed that VdR3e and homologous proteins formed three major clades, and the genetic distance between VdR3e and the proteins from the *Bipolaris* group (*Bipolaris maydis*, *Bipolaris maydis* ATCC 48331, and *Bipolaris victoriae FI3*) was the closest (Fig. 1A). The sequence alignment of VdR3e with the highest homology showed that VdR3e shares 15 conserved sites with these homologous proteins, and the conserved amino acids were mainly cysteine, proline, threonine, glycine, phenylalanine, tryptophan, serine, aspartic acid, and glutamine (see Fig. S1 in the supplemental material). These results indicate that VdR3e and homologous proteins are significantly different at the sequence level. The *VdR3e* gene was cloned, and the translated sequence was analyzed for protein structure. The program SignalP 5.0 (40) showed that VdR3e has a signal peptide in its N terminus, while TMHMM 2.0 (41) revealed that VdR3e had no transmembrane domain. The prediction program DiANNA 1.1 (42) showed that the six cysteine residues in VdR3e formed three pairs of disulfide bonds within VdR3e. Analyses of the VdR3e protein sequence by SMART (43) showed that VdR3e does not have typical domain characteristics (Fig. 1B). These results indicated that VdR3e is a secreted cysteine-rich protein.

### VdR3e is an effector triggering cell death and multiple other defense responses.

**FIG 2:**
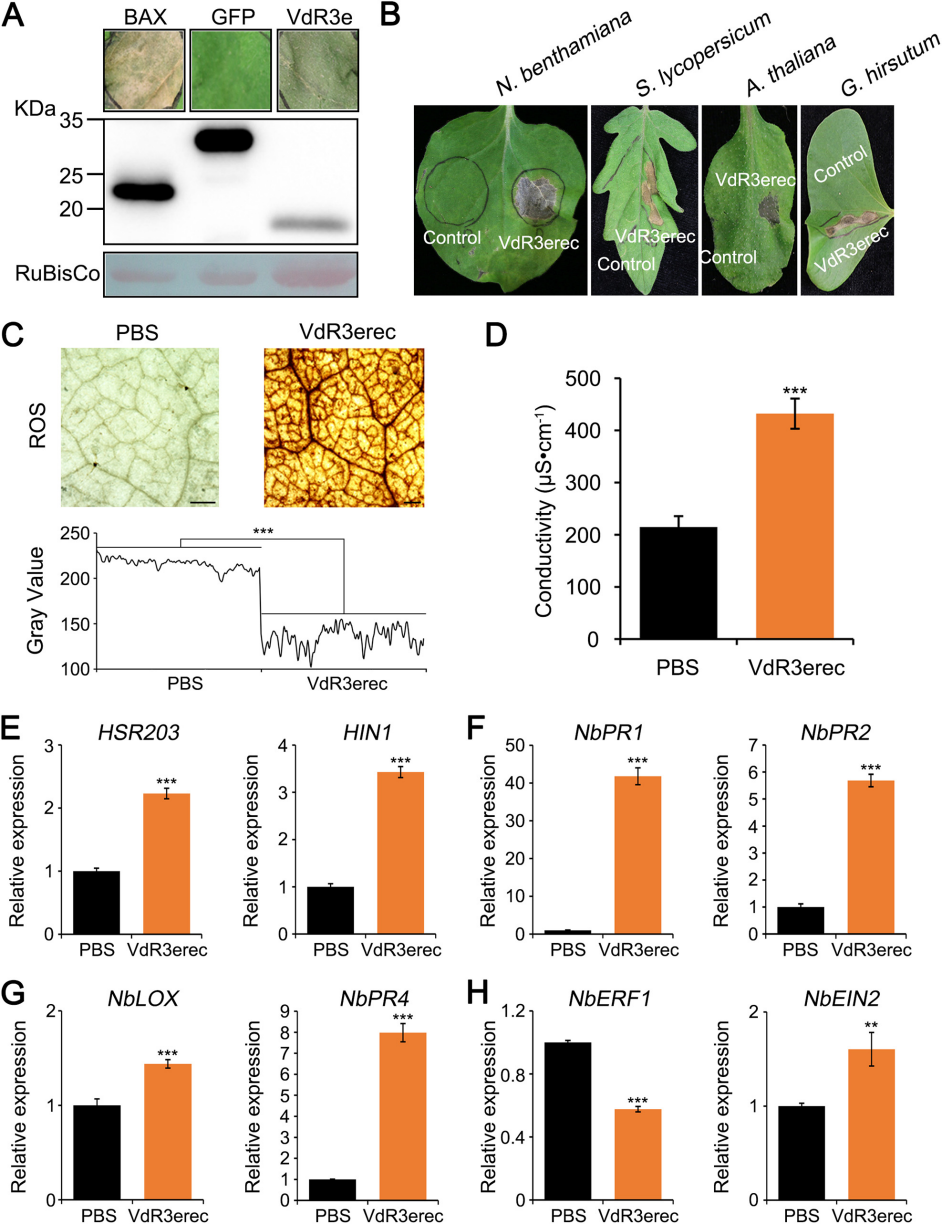
*V. dahliae* VdR3e induces cell death and multiple defense responses. (A) Analyses of induced cell death and immunoblotting. A. tumefaciens expressing VdR3e was infiltrated into 4-week-old N. benthamiana leaves to detect its activity in inducing cell death. GFP and BAX (Bcl-2-associated X protein) were used as negative and positive controls, respectively. Four-week-old N. benthamiana leaves were infiltrated with A. tumefaciens expressing target genes and harvested after 2 days. The harvested leaves were used to extract total proteins for Western blot analysis. RuBisCo protein was used as a total protein loading control. (B) Cell death-inducing activities in N. benthamiana, *S. lycopersicum*, *A. thaliana*, and *G. hirsutum* leaves were detected after infiltration with VdR3erec (50 μg/mL) and a PBS control. (C) Detection of ROS accumulation induced by VdR3e. Detection of ROS accumulation levels in N. benthamiana leaves after infiltration with VdR3erec (5 μg/mL) and PBS for 2 days. Scale bars, 500 μm. ImageJ was used to analyze the gray value. Values represent the means ± the standard errors (SE) of three independent samples. Asterisks (*), double asterisks (**), and triple asterisks (***) represent statistical significances at 0.01 < *P < *0.05, 0.001 < *P < *0.01, and *P < *0.001, respectively, based on unpaired Student *t* tests. (D) Detection of electrolyte leakage induced by VdR3e. The N. benthamiana leaves were infiltrated with VdR3erec (5 μg/mL) and PBS. The leaves were harvested 2 days later to assess electrolyte leakage. Values represent the averages of three independent measurements with three replicates each. Error bars represent the standard errors of the mean. Asterisks (*), double asterisks (**), and triple asterisks (***) represent statistical significance at 0.01 < *P < *0.05, 0.001 < *P < *.01, and *P < *0.001, respectively, based on unpaired Student *t* tests. (E to H) Detection of VdR3e-induced defense response-related gene expression. VdR3erec and a PBS control were infiltrated in N. benthamiana leaves, and the transcription levels of defense-related genes *HSR203* (E), *H1N1* (E), *NbPR1* (F), *NbPR2* (F), *NbLOX* (G), *NbPR4* (G), *NbERF1* (H), and *NbEIN2* (H) were quantified by RT-qPCR 2 days later. Values represent the means ± the SE of three independent samples. Asterisks (*), double asterisks (**), and triple asterisks (***) represent statistical significance at 0.01 < *P < *0.05, 0.001 < *P < *0.01, and *P < *0.001, respectively, based on unpaired Student *t* tests.

VdR3e was previously predicted to be an effector (39). To identify whether VdR3e can induce cell death, *VdR3e* was transiently overexpressed in Nicotiana benthamiana leaves. Cell death in N. benthamiana leaves was observed 5 days after agroinfiltration with Agrobacterium tumefaciens carrying the *VdR3e* overexpression construct (Fig. 2A). Immunoblotting analysis confirmed that VdR3e was expressed in N. benthamiana (Fig. 2A). To further confirm that VdR3e protein could induce cell death in N. benthamiana, the recombinant protein VdR3erec was expressed in a prokaryotic expression system and tested for cell death activity by infiltrating 50 μg/mL of the protein solution into N. benthamiana leaves. VdR3erec triggered cell death in N. benthamiana leaves 12 h after infiltration (Fig. 2B). To test the specificity of VdR3e, we infiltrated VdR3erec into various host leaves. VdR3erec induced cell death in Solanum lycopersicum (Ailsa Craig), *A. thaliana*, and Gossypium hirsutum (Fig. 2B). Thus, VdR3e can induce cell death in diverse hosts.

To further examine the role of *VdR3e* as an effector to induce plant immune responses, we analyzed reactive oxygen species (ROS) accumulation and electrolyte leakage in N. benthamiana leaves following infiltration with VdR3erec. Leaves infiltrated with phosphate-buffered saline (PBS) were used as a control. Compared to the control plants, plants infiltrated with VdR3erec displayed stronger ROS accumulation at 2 days postinoculation (Fig. 2C). Gray value analyses with ImageJ also confirmed this (Fig. 2C). The electrical conductivity of N. benthamiana leaves increased significantly 2 days after infiltration with VdR3erec compared to control plants (Fig. 2D). To investigate the molecular basis in which VdR3e triggers immunity, the expression of defense-related genes in VdR3erec-infiltrated N. benthamiana was monitored. The defense-related genes included (i) *HSR203* (44) and *H1N1* (45), marker genes for HR; (ii) *NbPR1* (46) and *NbPR2* (47), marker genes for salicylic acid (SA)-dependent defense; (iii) *NbLOX* and *NbPR4*, marker genes for jasmonic acid (JA)-dependent defense (48); and (iv) *NbERF1* and *NbEIN2*, marker genes for ethylene-dependent defense (49). Reverse transcription-quantitative PCR (RT-qPCR) analyses of the expression of *NbLOX*, *NbERF1*, and *NbEIN2* did not significantly change. However, the expression of *HSR203* and *H1N1* was slightly upregulated, and the expression of *NbPR1*, *NbPR2*, and *NbPR4* was significantly upregulated (Fig. 2E to H). VdR3e can therefore induce HRs and activate SA- and JA-dependent immune responses in N. benthamiana. These analyses indicated that VdR3e activates the plant’s immune system to cause cell death and is accompanied by the triggering of a variety of defense responses in plant cells.

### VdR3e localizes in the periphery of plant cells.

**FIG 3:**
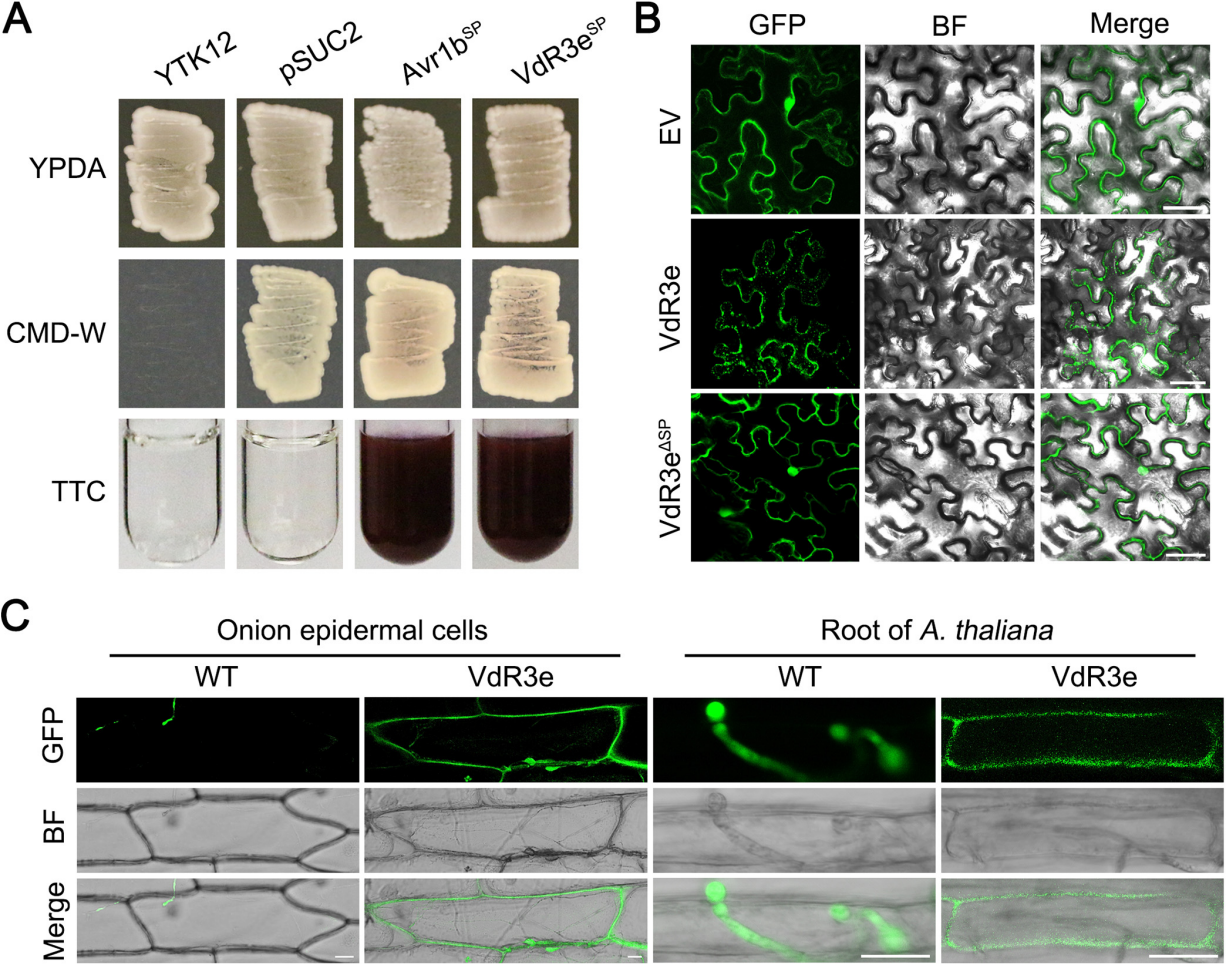
Subcellular localization of the *V. dahliae* VdR3e in plant tissues. (A) Validation of the signal peptide activity of *VdR3e* by yeast signal trap assay. The yeast strain YTK12 can grow on yeast extract peptone dextrose adenine (YPDA) medium and YTK12 containing the pSUC2 vector can grow on CMD-W medium. The fusion of VdR3e signal peptide with mature yeast invertase enables the invertase to be secreted. As a result, the color of TTC solution changes from colorless to red. Avr1b was used as a positive control. (B) Subcellular localization of VdR3e-GFP in N. benthamiana leaves. VdR3e and VdR3e^ΔSP^ were transiently expressed in N. benthamiana leaves by agroinfiltration. The pBin-GFP was used as a negative control. The fluorescence was scanned by Lecia TCS SP8 confocal microscopy system with an excitation wavelength at 488 nm and emission wavelength at 510 nm. Scale bars, 50 μm. (C) Subcellular localization of VdR3e-GFP in onion epidermal cells and roots of *A. thaliana*. The onion epidermis and roots of *A. thaliana* were immersed in the conidial suspension of HoMCLT overexpressing VdR3e-GFP and GFP for 20 min. The onion epidermal or *A. thaliana* tissues were incubated on water agar medium for 5 and 2 days, respectively. Finally, fluorescence was observed with confocal microscopy. Scale bars, 25 μm.

Indicative of its secretion, VdR3e has a signal peptide domain within the N-terminal amino acid residues 1 to 19 (Fig. 1B). To determine whether the signal peptide of VdR3e is functional, a yeast signal trap assay was performed (50). The VdR3e’s signal peptide region (VdR3e^SP^) was cloned into the yeast invertase vector pSUC2, and the recombinant vector was then transformed into yeast strain YTK12. As the positive-control signal peptide from *Phytophthora sojae*, apoplastic effector Avr1b (Avr1b^SP^) was utilized. VdR3e^SP^ can secrete sucrose converting enzyme to reduce triphenyltetrazolium chloride (TTC) to insoluble red triphenylformazan. In contrast, no change in color was observed in YTK12 or empty vector pSUC2 (Fig. 3A). This confirmed that VdR3e has a functional signal peptide that mediates its secretion. Furthermore, to detect the subcellular localization of VdR3e in N. benthamiana, VdR3e was fused with green fluorescent protein (GFP) at the C terminus. The recombinant constructs were transiently expressed in N. benthamiana leaves by agroinfiltration. The results showed that green fluorescence was observed mainly at the periphery of the N. benthamiana cell (Fig. 3B). Interestingly, transient expression of VdR3e lacking the signal peptide (*VdR3e^ΔSP^*) changed the location of the green fluorescence to random positions inside the cell (Fig. 3B). To further demonstrate that VdR3e is localized at the periphery of plant cell, VdR3e-GFP and GFP were overexpressed in the race 3 strain HoMCLT. Onion epidermis and *Arabidopsis* root infected by the HoMCLT strain overexpressing VdR3e-GFP and GFP were examined for green fluorescence. In both onion epidermis and *Arabidopsis* root, GFP as a control showed green fluorescence only in the conidia, while VdR3e specifically showed green fluorescence at the periphery of cells (Fig. 3C). These results indicated that VdR3e is localized at the periphery of plant cell during its interaction with plants.

### The distribution on the periphery of plant cells is necessary for VdR3e to induce immune responses and is BAK1 dependent.

**FIG 4:**
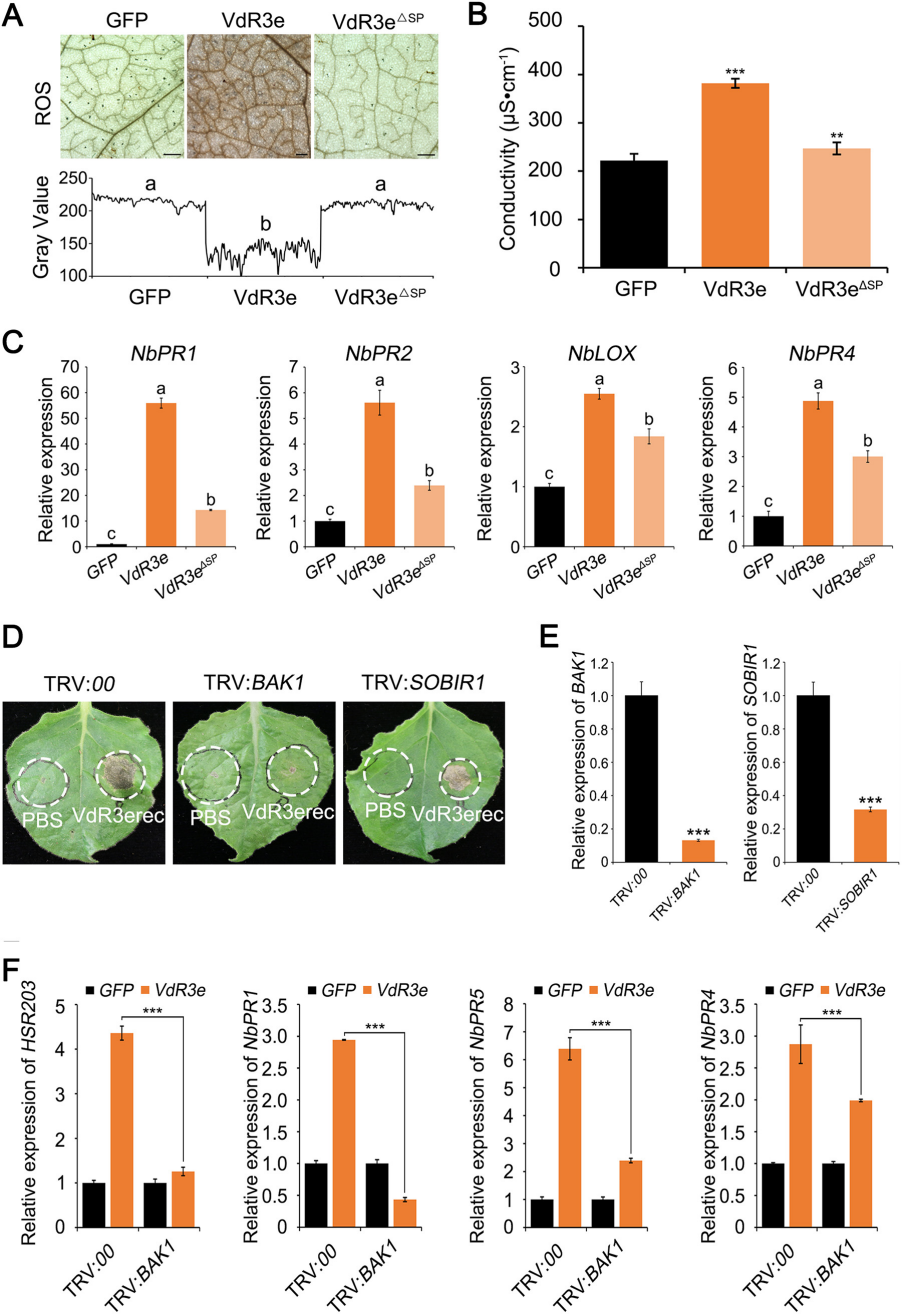
Analysis of the relationships between immune function induced by V. dahliae VdR3e and the peripheral extracellular localization and interactions with the N. benthamiana proteins BAK1 and SOBIR1. (A) ROS accumulation after transient expression of VdR3e, VdR3e^ΔSP^, and GFP control for 2 days. Scale bars, 500 μm. ROS accumulation levels in N. benthamiana leaves were measured after the transient expression of VdR3e, VdR3e^ΔSP^, and the GFP control for 2 days. ImageJ was used to analyze the gray value. Values represent the means ± the SE of three independent samples. Bars not sharing letters represent significant differences of the mean at *P < *0.05, based on unpaired Student *t* tests. (B) Detection of electrolyte leakage induced by VdR3e and VdR3e^ΔSP^. N. benthamiana leaves that transiently expressed VdR3e, VdR3e^ΔSP^, and the GFP control were tested for electrolyte leakage after 2 days. Values represent the averages of three independent measurements with three replicates each. Error bars represent standard errors of the mean. Asterisks (*), double asterisks (**), and triple asterisks (***) represent statistical significance at 0.01 < *P < *0.05, 0.001 < *P < *0.01, and *P < *0.001, respectively, based on unpaired Student *t* tests. (C) Detection of defense gene expression induced by VdR3e or VdR3e^ΔSP^. N. benthamiana leaves were harvested 2 days after transient expression of VdR3e, VdR3e^ΔSP^, and the GFP control. RT-qPCR was used to quantify the transcription levels of defense genes *NbPR1*, *NbPR2*, *NbLOX*, and *NbPR4*. Values represent the means ± the SE of three independent samples. Bars not sharing letters represent significant mean differences at *P < *0.05, based on unpaired Student *t* tests. (D) Verification of the relationship between N. benthamiana proteins BAK1 and SOBIR1 and the cell death-inducing function of VdR3e. Virus-induced gene silencing was performed on 3-week-old N. benthamiana. VdR3erec (50 μg/mL) and PBS were infiltrated in N. benthamiana leaves silenced with BAK1 and SOBIR1. (E) The expression levels of BAK1 and SOBIR1 in silenced plants were quantified by RT-qPCR. Values represent the means ± the SE of three independent samples. Asterisks (*), double asterisks (**), and triple asterisks (***) represent statistical significance at 0.01 < *P < *0.05, 0.001 < *P* < 0.01, and *P < *0.001, respectively, based on unpaired Student *t* tests. (F) Detection of transcription levels of VdR3e induced defense genes in silenced plants. Transient transformation of VdR3e was performed using *Agrobacterium* infiltration in N. benthamiana silenced leaves of BAK1 and SOBIR1. The expression levels of *HSR203*, *NbPR1*, *NbPR5*, and *NbPR4* were detected by RT-qPCR. Values represent the means ± the SE of three independent samples. Asterisks (*), double asterisks (**), and triple asterisks (***) represent statistical significance at 0.01 < *P < *0.05, 0.001 < *P* < 0.01, and *P* < 0.001, respectively, based on unpaired Student *t* tests.

Deletion of VdR3e signal peptide altered its subcellular localization (Fig. 3B). To investigate whether this change would affect VdR3e immune-inducing function, we analyzed ROS accumulation and electrolyte leakage in N. benthamiana leaves after agroinfiltration of the constructs encoding *VdR3e* and *VdR3e^ΔSP^*. *VdR3e*-expressing plants displayed strong ROS accumulation, whereas *VdR3e^ΔSP^*-expressing plants displayed no ROS accumulation just like the control plants (Fig. 4A). Compared to *VdR3e*-expressing plants, the conductivity of *VdR3e^ΔSP^*-expressing plants was significantly lower and was similar to the control (Fig. 4B). To further illustrate that the changes in VdR3e subcellular localization can affect its immune function, defense-related genes were detected after transient transformation of *VdR3e^ΔSP^*. The expression of SA pathway marker genes *NbPR1* and *NbPR2*, as well as JA pathway marker genes *NbLOX* and *NbPR4*, was significantly reduced in *VdR3e^ΔSP^*-expressing plants compared to *VdR3e*-expressing plants (Fig. 4C). The function of VdR3e induced immunity thus depended on its localization at the cell periphery. To test whether the function of VdR3e to induce cell death in N. benthamiana depends on BAK1 and SOBIR1, virus-induced gene silencing (VIGS) constructs were generated based on recombinant tobacco rattle virus (TRV) to target *BAK1* and *SOBIR1* expression in N. benthamiana. Twenty-one days after TRV inoculation, plants were agroinfiltrated with VdR3erec and a PBS control. VdR3e induced cell death in the *SOBIR1*-silenced plants, as well as in the control plants. Interestingly, VdR3e failed to induce cell death in the *BAK1*-silenced plants (Fig. 4D). RT-qPCR analysis confirmed that the transcript levels of *BAK1* and *SOBIR1* were significantly reduced to 10 and 30% of the control (Fig. 4E). To further confirm that VdR3e-triggerd defense responses require BAK1, we expressed *VdR3e* transiently in *BAK1*-silenced plants and monitored the expression of defense-related genes. The expression of defense-related genes decreased significantly (Fig. 4F). The results indicate that the coreceptor BAK1 is required for VdR3e-triggered cell death in N. benthamiana. Collectively, VdR3e-induced defense responses depends on its peripheral extracellular localization and the membrane receptor BAK1.

### VdR3e shows host dependency.

**FIG 5:**
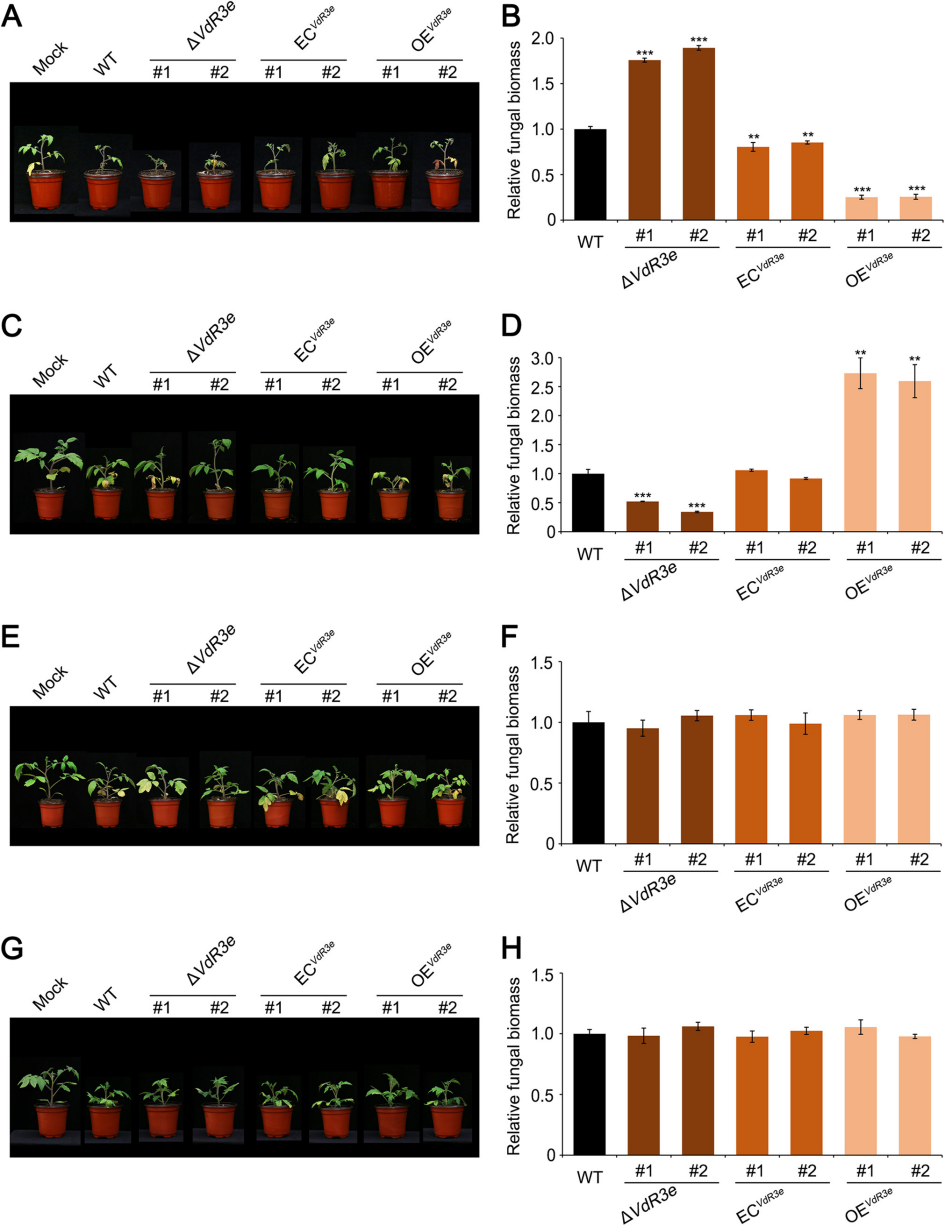
Analysis of the virulence function of VdR3e from Verticillium dahliae in tomato. (A, C, E, and G) Phenotypes of the *S. lycopersicum* cultivars Ailsa Craig, Zhongfan No.310, Zhongfan No.144, and Moneymaker inoculated, respectively, with ΔVdR3e-HoMCLT, EC^VdR3e^-HoMCLT, and OE^VdR3e^-HoMCLT strains. Two-week-old plants of each cultivar were inoculated by the root-dipping method with ΔVdR3e-HoMCLT, EC^VdR3e^-HoMCLT, and OE^VdR3e^-HoMCLT strains, the wild type (HoMCLT), or sterile water (Mock) as a control. The separate transformants were characterized for each mutant. Symptoms were recorded 21 days after inoculation. (B, D, F, and H) Detection of the fungal biomass of the ΔVdR3e-HoMCLT, EC^VdR3e^-HoMCLT, and OE^VdR3e^-HoMCLT strains after inoculating *S. lycopersicum* cultivar Ailsa Craig, Zhongfan No.310, Zhongfan No.144, and Moneymaker. Three weeks after inoculation, the roots and stems of the *S. lycopersicum* were harvested. The genomic DNA of the harvested tissue was extracted, and the fungal colonization of V. dahliae in the *S. lycopersicum* roots was detected by quantitative PCR. Values represent the means ± the SE of three independent samples. Asterisks (*), double asterisks (**), and triple asterisks (***) represent statistical significance at 0.01 < *P < *0.05, 0.001 < *P* < 0.01, and *P < *0.001, respectively, based on unpaired Student *t* tests. (A and B) Ailsa Craig; (C and D) cultivar Zhongfan No.310; (E and F) cultivar Zhongfan No.144; (G and H) cultivar Moneymaker.

The role of *VdR3e* in pathogenicity was examined by additional analyses of the *VdR3e* deletion strain HoMCLT and its complemented strains (39), along with the overexpression transformants that were generated in this study. The pathogenicity of these strains was tested on three host plants—N. benthamiana, *G. hirsutum*, and *S. lycopersicum*—using a root-dip method of inoculation (21, 51). In N. benthamiana, at 21 days postinoculation, tobacco inoculated with the wild-type HoMCLT strain showed symptoms of yellowing and wilting, and N. benthamiana inoculated with the deletion strains and overexpression strains showed comparable symptoms of Verticillium wilt (see Fig. S2A). The colonization of V. dahliae in N. benthamiana roots was assessed by quantitative PCR. There was no significant difference in the biomass of V. dahliae in N. benthamiana roots inoculated with wild-type, VdR3e deletion mutant, complemented, and VdR3e-overexpressing strains (see Fig. S2B). VdR3e in HoMCLT thus had no effect on tobacco. In *G. hirsutum*, the susceptible cultivar Junmian No.1 was healthy 21 days after inoculations with HoMCLT (see Fig. S2C) and did not exhibit any change when inoculated with knockout, complemented, or overexpressing strains (see Fig. S2D). These results indicated that VdR3e cannot contribute to the pathogenicity of HoMCLT in cotton. However, in *S. lycopersicum*, HoMCLT strains caused the tomato cultivar Ailsa Craig to wither and dwarf 21 days after inoculation. Interestingly, compared to Ailsa Craig inoculated with the wild-type strains, Ailsa Craig inoculated with the VdR3e deletion strains were more wilted and dwarfed, while those inoculated with the complemented strains exhibited the same degree of disease as the wild type (Fig. 5A). Those inoculated with the VdR3e-overexpressing strains showed mild disease symptoms (Fig. 5A). The results of quantitative PCR on V. dahliae biomass in infected Ailsa Craig roots were consistent with the phenotype (Fig. 5B). Thus, VdR3e acts as a negative regulator of HoMCLT pathogenicity in tomato cultivar Ailsa Craig, and VdR3e exhibits host specificity. To further demonstrate the host specificity of VdR3e, we also tested the virulence function of VdR3e on three other tomato cultivars, including cultivar Zhongfan No.310, cultivar Zhongfan No.144, and cultivar Moneymaker. Cultivar Zhongfan No.310 plants displayed lower dwarfing and wilting when inoculated with the VdR3e deletion strains than the wild-type strain and recovered high virulence when inoculated with the VdR3e-complemented transformants (Fig. 5C and D). In contrast, in Zhongfan No.144 and Moneymaker cultivars, VdR3e did not show a virulence function (Fig. 5E to H). The results suggested that VdR3e diverged in its role in pathogenicity on the different tomato cultivars, i.e., the role of VdR3e in pathogenicity is host and cultivar dependent.

### VdR3e has no effect on the growth and morphology of V. dahliae.

**FIG 6:**
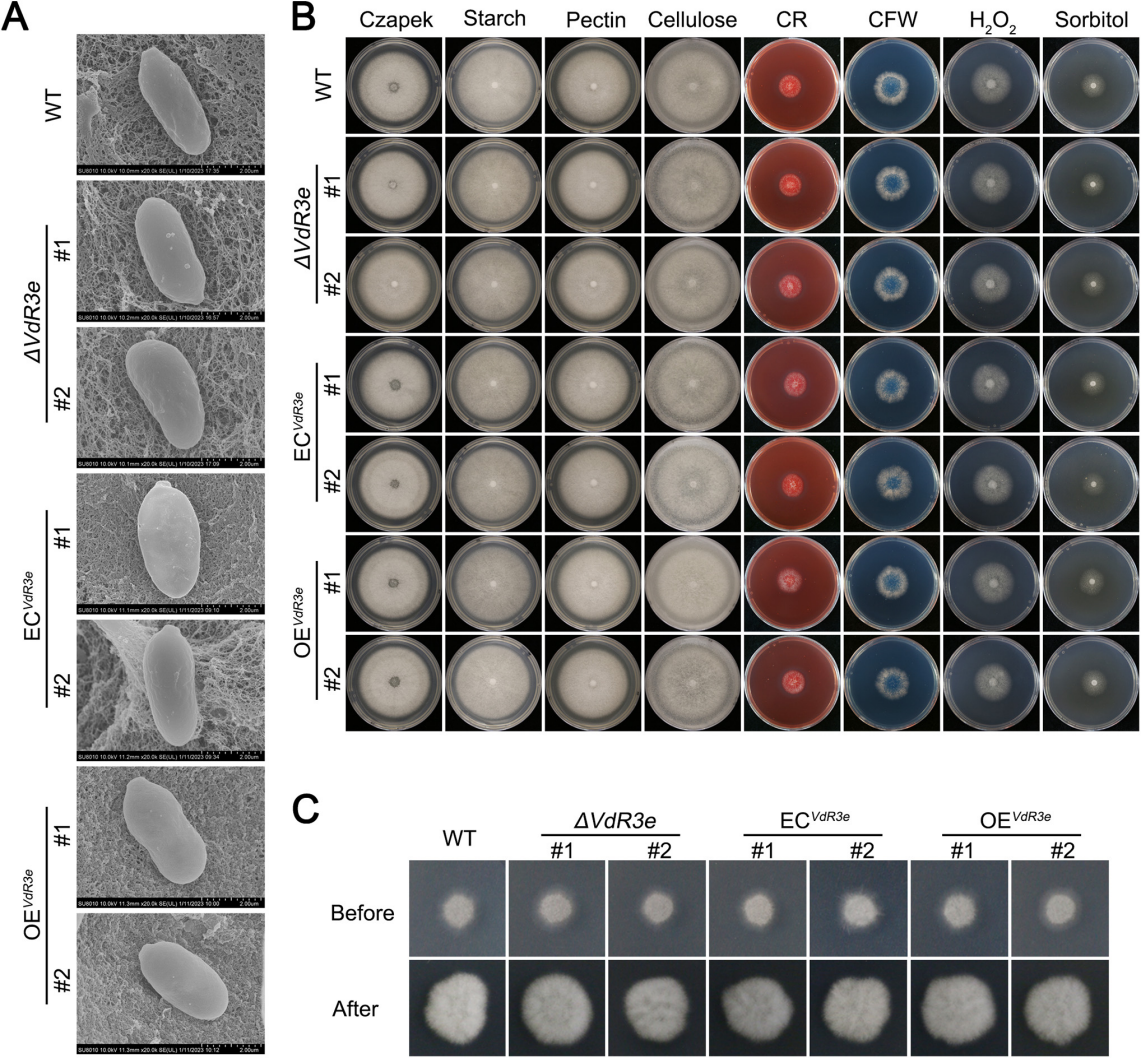
Growth phenotypes of ΔVdR3e-HoMCLT, EC^VdR3e^-HoMCLT, and OE^VdR3e^-HoMCLT strains. (A) Observations of the ΔVdR3e-HoMCLT, EC^VdR3e^-HoMCLT, and OE^VdR3e^-HoMCLT conidia of the ΔVdR3e-HoMCLT, EC^VdR3e^-HoMCLT, and OE^VdR3e^-HoMCLT strains by scanning electron microscope. Scale bars, 2 μm. (B) Analysis of effects of VdR3e on the utilization of different carbon sources by HoMCLT and the growth of HoMCLT under stress. The conidial suspensions of V. dahliae strains were prepared at a concentration of 5 × 10^6^ conidia/mL. Then, 2-μL conidial suspensions were cultured on different carbon sources and different media containing stressors at 25°C. The growth phenotype was photographed 7 days later. HoMCLT (WT) was used as a control. Two separate transformants were characterized for each mutant. (C) Effect of VdR3e on the penetrating ability of HoMCLT. The mutant strains were cultured in cellophane-lined minimal medium to simulate penetration of cell walls. The mutant strain was cultured on cellophane-lined minimal medium for 3 days. After the 3-day period, the cellophane was removed, and the culture continued. The growth phenotype was photographed after 5 days. Two separate transformants were characterized for each mutant.

In order to investigate whether VdR3e affects the normal growth of HoMCLT, VdR3e deletion, complemented, and VdR3e-overexpressing strains were observed by using a scanning electron microscope. The presence or absence of VdR3e had little effect on the morphology of HoMCLT conidia (Fig. 6A). Furthermore, VdR3e affected HoMCLT’s ability to use different carbon sources on Czapek medium, where sucrose was replaced by starch, pectin, and cellulose. We also tested the colony diameters of the mutant strains on Congo red and calcofluor white medium to simulate whether it participated in response to cell wall integrity. The mutant strains were cultured on Czapek medium supplemented with H_2_O_2_ and sorbitol to test whether VdR3e affected the growth of HoMCLT under oxidative stress and hyperosmotic stress. Compared to wild-type strains, none of the mutant strains changed significantly under any of the experimental conditions described above (Fig. 6B; see also Fig. S3). Finally, we also tested whether VdR3e deletion affected the penetration of HoMCLT, and the mutant strains penetrated cellophane, as well as the wild-type strains (Fig. 6C). VdR3e did not affect the morphology and growth of HoMCLT under normal conditions, nor did it affect the growth under oxidative or hyperosmotic stress. These results indicated that VdR3e acts as a *bona fide* effector that does not affect growth and morphology but also does not affect penetration.

## DISCUSSION

Research on different pathogenic races has remained a topic of interest in plant pathology. *Verticillium* wilt resistance in tomato was first reported to be mediated by *Ve* in 1951 (11). With the large-scale planting of the resistant cultivar Pakmor, *Ve1*-mediated resistance to race 1 of V. dahliae was quickly overcome by race 2 (13, 18). Race 2 and an additional race of V. dahliae were characterized based on the responses of differential tomato cultivars by Usami et al. (14). Through rapid developments in genome sequencing and comparative genomics, the *avr* gene *Ave1* in race 1 of V. dahliae and the *avr* gene *Av2* in race 2 of V. dahliae were identified (29, 38). Later, through comparative analysis of the genomes of strains representing the three races of V. dahliae, the secreted protein VdR3e was found only in race 3 and was demonstrated to encode the *avr* gene of race 3 by genetic experiments (39). However, there remained a lack of understanding as to how *VdR3e*, as an *avr* gene, mediates the immune responses. The study here systematically investigated the characteristics of VdR3e-mediated immune responses. VdR3e is an effector that can induce cell death by activating a variety of defense responses. Moreover, the ability to cause cell death is dependent on peripheral extracellular localization and is also BAK1 dependent. The function of VdR3e was host and cultivar dependent, and this is likely caused by the recognition of VdR3e by different receptors in different hosts/cultivars.

Previous studies suggested that Ave1 was homologous to the plant natriuretic peptide family and was transferred from plants to *Verticillium* by horizontal gene transfer (29); other examples of horizontal gene transfer have been characterized in V. dahliae (52). Our phylogenetic analysis revealed that VdR3e and its homologous proteins formed three major branches and had a low sequence similarity, suggesting that the VdR3e gene may have been acquired through at least three independent horizontal gene transfers (Fig. 1A; see also Fig. S1). Although the exact donor of the *VdR3e* gene is not yet known, phylogenetic and sequence alignment results indicate that the *VdR3e* gene is derived most likely from the fungal genus *Bipolaris*.

According to the gene-for-gene model, only in the presence of a dominant resistance gene (*R*) in the host plant, the pathogen expresses the corresponding dominant avirulence gene (*Avr*), and as a result, the host plant exhibits resistance (53). *Ve1* mediates the resistance of race 1 of V. dahliae, the product of which recognizes Ave1 and causes *Ve1*-carrying tomatoes to exhibit resistance (29). The coexpression of Ave1 and Ve1 can activate HRs in tobacco and tomato (30). Similarly, we found that the transient expression of VdR3e in tobacco can also induce HRs resulting in cell death (Fig. 2A to D). Ave1 can induce the expression of several defense genes, including *PR1*, *PR2*, and peroxidase (32). Overexpression of VdR3e in plants also activated the expression of some defense genes. In addition to *PR1* and *PR2* in the SA pathway, VdR3e also activated the HR and JA pathway marker genes (Fig. 2E to H). In contrast, the mutation or loss of the *R* gene in plants or the *Avr* gene in the pathogen causes the plant to change from resistance to susceptibility (53). Ave1 showed significant pathogen virulence to tomatoes when the plants did not carry *Ve1* (29). Avirulence genes are considered bifunctional effectors, which play an important role in determining both the avirulence and the pathogenicity of pathogens. VdR3e acts as an avirulence factor in Ailsa Craig and as a virulence factor in cultivar Zhongfan No.310 (Fig. 5A to D). Interestingly, VdR3e did not affect the virulence of HoMCLT on tobacco and on tomato cultivars Zhongfan No.144 and Moneymaker (Fig. 5E to H; see also Fig. S2A and B). This is presumably the result of the coproduction of VdR3e in plants as a virulence factor on the one hand and as an immune activator on the other.

To resist the invasion of pathogens, plants have evolved two layers of innate immune system. Pathogen surfaces may secrete many specific substances (proteins, polysaccharides, etc.) that are not present in the host plant, which is called the pathogen associated molecular pattern (PAMP) (54, 55). PAMPs are recognized by pattern recognition receptors (PRRs) on the surfaces of plant cells, triggering the plant’s first layer of immune responses: the PAMP-triggered immune response (PTI). The second layer of plant immunity is the effector-triggered immune response (ETI) triggered by nucleotide-binding, leucine-rich repeat (NLR) proteins acting as intracellular immune receptors to recognize the pathogen effectors (56). Most of the *avr* gene products are recognized by the NLR type receptor proteins in the cell (57). For example, the effector ATR1 (Arabidopsis thaliana recognized 1) of the pathogen *Hyaloperonospora arabidopsidis* can interact directly with RPP1 (*Arabidopsis* NLR recognition of *Peronospora parasitica* 1) inside the cell to trigger immune responses (58). *Arabidopsis* resistance protein RPS2 triggers immune signals by sensing the conformation change of RIN4 (*rpm1* interacting protein 4) by AvrRpt2, an effector in Pseudomonas syringae type III (59). *Arabidopsis* RRS1 is recognized by P. syringae type III effector AvrRps4, along with RPS4, by binding to an integrated C-terminal WRKY transcription factor domain, which induces an RPS4-dependent immune response through an interaction between the RRS1 and WRKY domain inside cell (60). However, there is also evidence that not all *Avr* genes are recognized by *R* gene products inside host cell. *Ve1* encodes an extracellular leucine-rich repeat receptor-like protein (eLRR-RLP), suggesting that it is a plasma membrane protein (21). Recognition of Ave1 by Ve1 is likely to occur in the plasma membrane, although no direct evidence of this has been found to date. Normally, interaction with BAK1 is considered to be evidence of PTI. The work of Fradin et al. (21) demonstrated that the recognition of Ave1 by Ve1 depends on BAK1. Thus, all of the results point to Ave1 as a potential PAMP. In our study, the *avr* gene *VdR3e* localized at the periphery of plant cell (Fig. 3B), and the function of VdR3e to induce immunity depended on its peripheral extracellular localization (Fig. 4A to C). We also demonstrated that the function of VdR3e in inducing immunity is dependent on BAK1 (Fig. 4D). These results suggest that VdR3e may also be a potential PAMP. The contribution of PAMP to virulence is not certain. The pathogenicity of specific Pseudomonas syringae pv. *tabaci* flagellin mutants to the host is reduced (61–63). CBEL as a PAMP in N. benthamiana and *Arabidopsis* can induce HRs but does not contribute to virulence (64, 65). This might explain why VdR3e has no effect on the virulence of V. dahliae race 3 on tobacco, cultivar Zhongfan No.144 and Moneymaker. There is growing evidence that simply dividing immune-related molecules into PAMP and effector, or PRR and R proteins, is becoming obsolete (57). Therefore, *VdR3e* can be recognized as an *avr* gene by unknown resistance gene to co-confer host resistance and also can be recognized as PAMP by unknown membrane receptors to activate defense responses. BAK1 usually mediates the LRR-RLP- and/or LRR-RLK-induced immune responses, while SOBIR1 usually induces the LRR-RLP-induced immune responses (37). Thus, we hypothesize that the unknown resistance protein interacting with VdR3e in the host is an LRR-RLK.

In conclusion, our study demonstrated, through transient expression of VdR3e in tobacco leaves, that VdR3e is a cell death activator dependent on its peripheral extracellular localization. VdR3e as a PAMP induced immune responses in a BAK1-dependent manner. In addition, VdR3e divergence in pathogenicity functions in tobacco, cotton, and tomato—and even among the different cultivars of tomato. These results suggest that *VdR3e*, as an *avr* gene, can play a role in both immunity and virulence.

## MATERIALS AND METHODS

### Bioinformatics analysis.

A phylogenetic tree was constructed by using MEGA5.05 and a maximum release method with VdR3e and homologous sequences in fungi (see Table S1 in the supplemental material). There were 1,000 rapid bootstrap approximations. The prediction of conserved sites was achieved through the online web program ESPript 3.0 (http://www.ipbs.fr/ESPript) (66). The signal peptide, transmembrane domains, disulfide bonds, and conserved domains of VdR3e were predicted based on the online programs SignalP 5.0 (http://www.cbs.dtu.dk/services/SignalP/) (40), TMHMM 2.0 (http://www.cbs.dtu.dk/services/TMHMM/) (41), DiANNA 1.1 (http://bioinformatics.bc.edu/clotelab/DiANNA/) (42), and SMART (http://smart.embl.de/) (43).

### Fungal culture and plant growth.

The race 3 V. dahliae strain HoMCLT and the mutant strain of HoMCLT were cultured on potato dextrose agar (PDA) or in liquid complete medium for 7 days at 25°C. Agrobacterium tumefaciens GV3101 for transient-expression experiments in plants was cultured in Luria-Bertani medium at 28°C for 2 days. N. benthamiana and A. thaliana were grown for 4 weeks for transient-expression experiments, and N. benthamiana was grown for 3 weeks for pathogenicity assays. Tomato (including cultivar Ailsa Craig, Zhongfan No.310, cultivar Zhongfan No.144, and cultivar seedlings Moneymaker, provided by the Institute of Vegetables and Flowers, CAAS) were grown for 2 weeks for pathogenicity assays and for 4 weeks for transient-expression experiments. All of the plants in this study were grown in the greenhouse at 25°C during 16-h/8-h day/night periods.

### Plasmid construction and preparation.

For transient-expression experiments, the tested genes were cloned from HoMCLT cDNA using the primers in Table S2, including *VdR3e* and *VdR3e^ΔSP^*. These sequences were cloned to the PVX vector pGR107 and transformed into the A. tumefaciens strain GV3101. For yeast signal sequence trap system experiments, the sequence encoding the signal peptide of VdR3e was cloned into the vector pSUC2 (67) to form the recombinant construct pSUC2::VdR3e^SP^. For the subcellular localization experiment, *VdR3e* and *VdR3e^ΔSP^* were cloned into the vector pBin after fusion of GFP to construct pBin::*VdR3e* and pBin::*VdR3e^ΔSP^*. For virus-induced gene silencing experiment, the genes BAK1 and SOBIR1 were cloned from N. benthamiana and cloned into the vector pTRV2 to generate pTRV2::*BAK1* and pTRV2::*SOBIR1*. In order to obtain the mutant material of V. dahliae, VdR3e was cloned into the vector pCOM. The recombinant constructs pCOM::*VdR3e* and pCOM::*VdR3e*::*GFP* were used to produce mutant strains of HoMCLT by an *Agrobacterium*-mediated transformation method described previously (50). Positive transformants were verified by RT-qPCR using the primers listed in Table S2.

### *Agrobacterium* and protein infiltration assays.

Agrobacterium tumefaciens carrying the target genes were resuspended in 10 mM MES, 10 mM MgCl_2_, and 0.2 mM acetosyringone (pH 6 to 7) to an optical density at 600 nm of 1.0. The A. tumefaciens suspensions were incubated at 28°C for 3 h. Concentrations of 50 or 5 μg/mL protein VdR3erec were used for protein infiltration experiments. Both A. tumefaciens suspensions and protein were infiltrated in 4-week-old *N. benthamiana* leaves with a 1-mL syringe. Each assay was performed on three leaves from three individual plants and repeated at least three times.

### Immunoblotting.

Immunoblotting was used to verify the amount of protein produced by transient expression of the target genes in *N. benthamiana*. Two days after infiltration, *N. benthamiana* leaves transiently transformed with target genes were harvested. Total protein was extracted with a P-PER plant protein extraction kit (Thermo Scientific) and a protease inhibitor cocktail kit (Thermo Scientific) according to the manufacturer’s instructions. The proteins were separated using 15% SDS-PAGE gels, and transient expression of protein was assessed using anti-HA antibody (Sigma) and detected with a Pierce ECL Western blotting substrate (Thermo Scientific).

### ROS activity and electrolyte leakage.

As described previously (68), ROS accumulation was visualized using 3,3′-diaminobenzidine (DAB) solution (Solarbio). *N. benthamiana* leaves were examined 2 days after agroinfiltration. Electrolyte leakage assays were performed as described by Oh et al. (69). The Probe LE703 (Mettler-Toledo) was used to measure ion conductivity. All experiments were repeated three times.

### Yeast signal sequence trap system.

The yeast signal sequence trap system was used as described previously (50) to test whether VdR3e’s signal peptide is functional. Briefly, the signal peptide region of VdR3e was cloned into the yeast invertase vector pSUC2, and then the recombinant vector was transformed into yeast YTK12. Positive inverters can grow on CMD-W (without tryptophan) medium. The yeast strain YTK12 containing functional signal peptide can change TTC from colorless to red.

### Subcellular localization assays.

In order to detect the subcellular localization of VdR3e, we constructed pBin::*VdR3e*, pBin::*VdR3e^ΔSP^*, and pCOM::*VdR3e*::*GFP*. The vectors pBin::*VdR3e* and pBin::*VdR3e^ΔSP^* were transformed into A. tumefaciens GV3101 and agroinfiltrated into the leaves of 4-week-old *N. benthamiana*. The leaves were harvested 2 days after infiltration. The onion epidermis and the root of the *A. thaliana* were immersed in HoMCLT conidial suspension overexpressing VdR3e-GFP for 20 min and then cultured on water agar medium. The onion epidermis was harvested after 5 days and the root of *A. thaliana* after 2 days. The fluorescence of all harvested materials was scanned by using a Leica TCS SP8 confocal microscopy system with an excitation wavelength at 488 nm and an emission wavelength at 510 nm.

### RNA extraction and analysis of gene expression.

Total RNA was isolated using a EASYspin Plus Plant RNA kit (Aidlab Biotechnologies, Beijing, China). First-strand cDNA was synthesized according to the instructions of TransScript one-step gDNA removal and cDNA synthesis supermix (TransGen Biotech, Beijing, China). RT-qPCR was performed under the following conditions: there was an initial 95°C denaturation step for 10 min, followed by denaturation for 15 s at 95°C, annealing for 30 s at 60°C, and extension for 30 s at 72°C for 40 cycles using *Taq* Pro Universal SYBR qPCR Master Mix (Vazyme Biotech, Nanjing, China). N. benthamiana elongation factor 1-α (*NbEF-1α*) was used as an endogenous reference. The primers used to detect the transcripts are listed in Table S2 in the supplemental material. The relative expression levels of genes were measured using the 2^–ΔΔ^*^CT^* method in three independent experiments (70). Unpaired Student *t* tests were performed to determine statistical significance.

### Virus-induced gene silencing in *N. benthamiana*.

VIGS was conducted using recombinant tobacco rattle virus (TRV) methods as described previously (71). The recombinant vectors (pTRV2::*BAK1* and pTRV2::*SOBIR1*) were constructed and transferred into A. tumefaciens GV3101. A. tumefaciens containing recombinant vectors and A. tumefaciens containing pTRV1 were mixed in a ratio of 1:1. The mixture was infiltrated into the two lowermost leaves of 3-week-old *N. benthamiana* plants. The phytoene desaturase (*PDS*) gene was used to assess the time of gene silencing (72). The silencing efficiency of *NbBAK1* and *NbSOBIR1* was detected by RT-qPCR. Each experiment was repeated three times.

### Phenotypic characterization.

Czapek medium was used as the base medium to simulate the growth of wild-type, knockout, complement, and overexpressed mutant strains under different carbon sources (carbon sucrose was supplemented by sucrose, starch, pectin, and cellulose) and stress conditions (add 1 M H_2_O_2_ and 1.2 M sorbitol). Conidial suspensions of V. dahliae strains were prepared at 5 × 10^6^ conidia/mL. Then, 2-μL conidial suspensions were cultured on the medium described above at 25°C. After 7 days, the colony growth phenotype was observed, and the colony growth diameter was measured. Each strain was cultured on three plates, and the experiment was repeated three times. Unpaired Student *t* tests were performed to determine statistical significance.

### *V. dahliae* infection and virulence assays.

Root-dip inoculation was used to test the pathogenicity of *G. hirsutum*, *N. benthamiana*, and *S. lycopersicum*, as previously described (21, 51). To visualize the fungal biomass, the roots from the four plants were harvested at 21 days after inoculation and used to extract genomic DNA. Genomic DNA was extracted according to the instructions of a DNAsecure plant kit (Tiangen Biotech, Beijing, China). Quantitative PCR was performed using the *Taq* Pro Universal SYBR qPCR Master Mix (Vazyme Biotech) to detect fungal colonization in plant roots. The V. dahliae ITS sequence was used to quantify fungal biomass, and the cotton *18S rRNA* gene, N. benthamiana elongation factor 1-α, and the tomato *actin* gene served as endogenous plant controls. The primers used are listed in Table S2. Unpaired Student *t* tests were performed to determine statistical significance.

### Data availability.

Sequence data from this article can be found in the GenBank data libraries. The accession numbers are displayed in Table S1 in the supplemental material.
