# Supplementary material for: Functional Characterization of Verticillium dahliae Race 3-Specific Gene VdR3e in Virulence and Elicitation of Plant Immune Responses
Source: Microbiol Spectr. 2023 Jun 28;11(4):e01083-23. doi: 10.1128/spectrum.01083-23 (PMC10434166; doi:10.1128/spectrum.01083-23)
Supplement: Supplemental file 1 — Fig. S1-S3. Download spectrum.01083-23-s0001.pdf, PDF file, 0.7 MB [file spectrum.01083-23-s0001.pdf]

Supplementary Material

Supplementary file 1

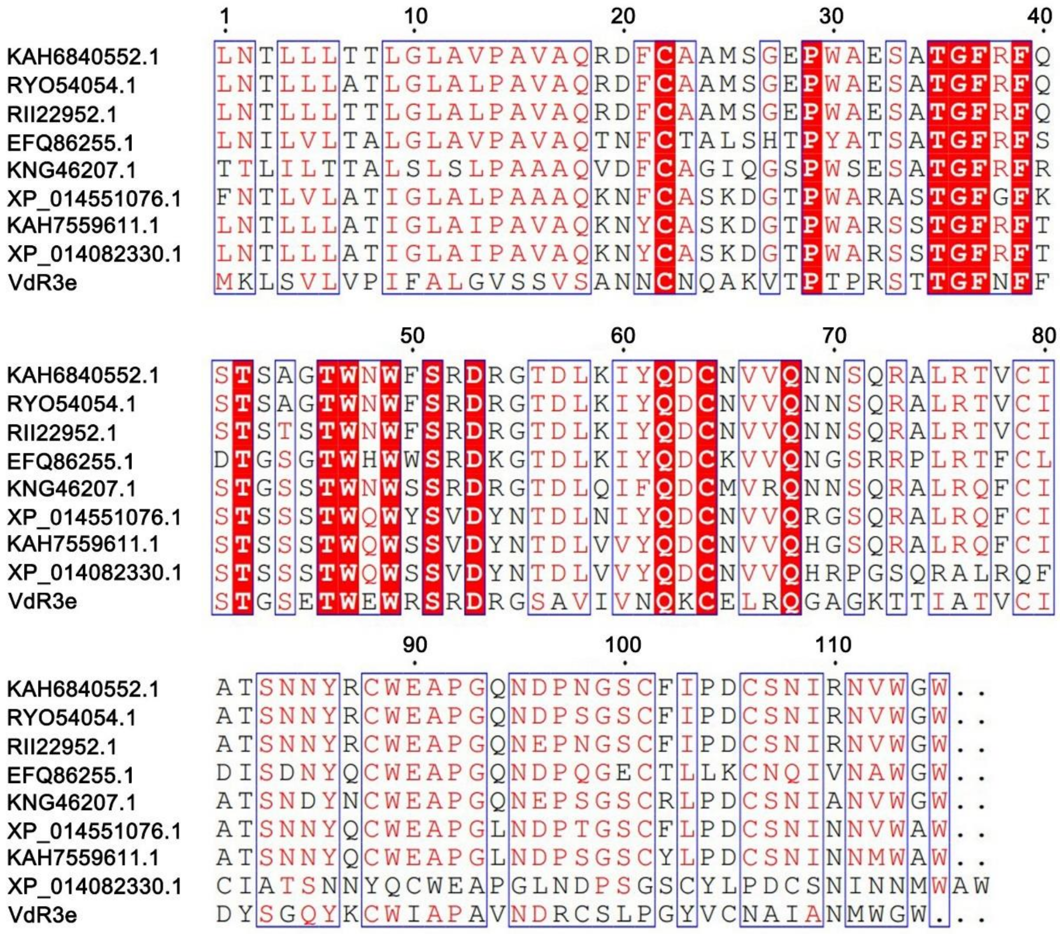

Fig S1 Multiple sequence alignment of VdR3e and homologous proteins. Multiple sequence alignments of VdR3e and its homologous proteins on the same evolutionary clade (KAH6840552.1, RYO54054.1, RII22952.1, EFQ86255.1, KNG46207.1, XP_014551076.1, KAH7559611.1 and XP_014082330.1) were performed based on the online tool ESPript. Conserved residues were written in white in red box. Red characters represent similarity in a group. A blue frame indicates similarity across groups.

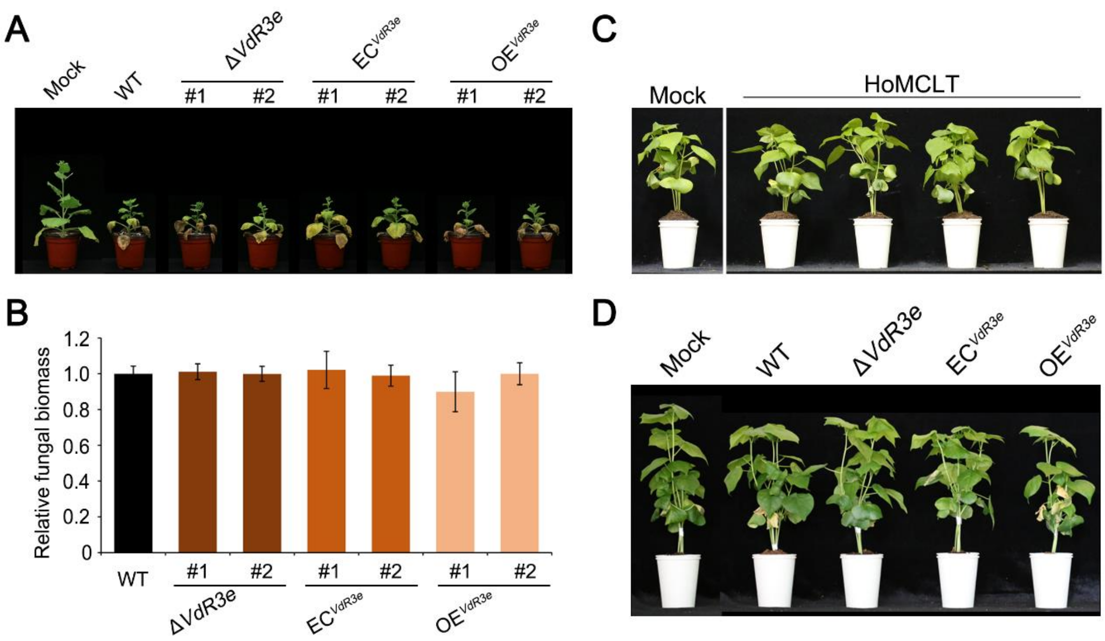

Fig S2 Analysis of the virulence function of VdR3e from *Verticillium dahliae* on *N. benthamiana* and cotton. (A) Phenotypes of the *N. benthamiana* plants inoculated with ΔVdR3e-HoMCLT, EC^{VdR3e}-HoMCLT and OE^{VdR3e}-HoMCLT strains. The 3-week-old *N. benthamiana* plants were inoculated by a root-dipping method with ΔVdR3e-HoMCLT, EC^{VdR3e}-HoMCLT and OE^{VdR3e}-HoMCLT strains, the wild type (HoMCLT) and sterile water (Mock) as control. Individual transformants were characterized for each mutant. Symptoms were recorded 21 days after inoculation. (B) Detection of the fungal biomass of the ΔVdR3e-HoMCLT, EC^{VdR3e}-HoMCLT and OE^{VdR3e}-HoMCLT strains following inoculations of *N. benthamiana* plants. Three weeks after inoculation, the roots and stems of the *N. benthamiana* were harvested. The genomic DNA of the harvested tissue was extracted and the fungal colonization of *V. dahliae* in the *N. benthamiana* roots were detected by quantitative PCR. Values represent the means ±*SE* of three independent samples. Statistical significance was calculated by an unpaired Student's *t* tests. (C) Detection of pathogenicity of HoMCLT on cotton. Cultivar Junmian No. 1 was selected for testing in this experiment. Cotton was inoculated with HoMCLT by the root-dip inoculation just after the second true leaf appeared. The phenotypes were photographed 3 weeks later. (D) Phenotypes of the ΔVdR3e-HoMCLT, EC^{VdR3e}-HoMCLT and OE^{VdR3e}-HoMCLT strains inoculated on cotton. After 3 weeks, cotton plants inoculated with the mutant strains were photographed.

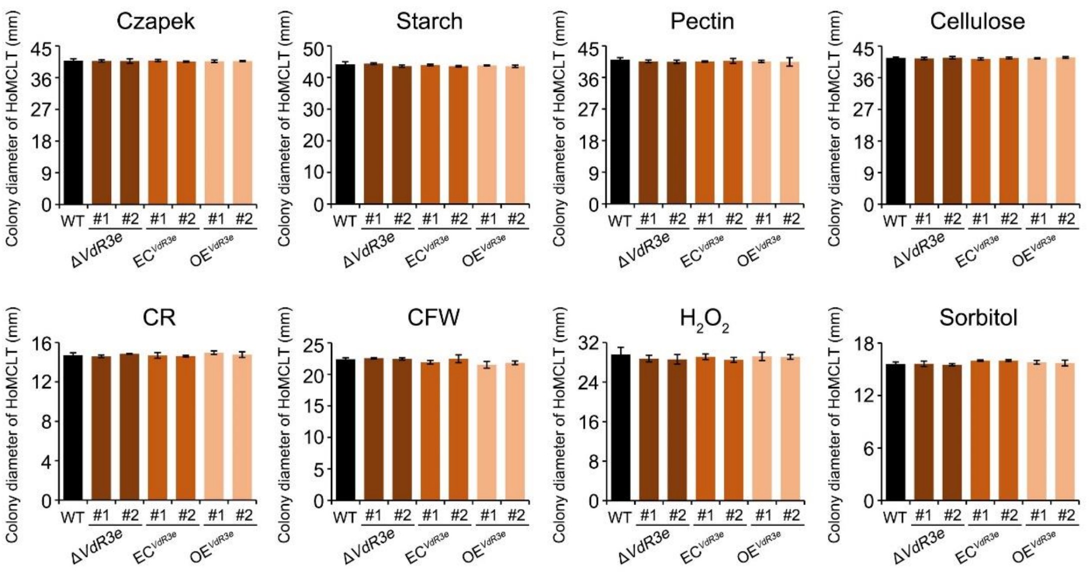

Fig S3 The radial growth of the ΔVdR3e-HoMCLT, EC^{VdR3e}-HoMCLT and OE^{VdR3e}-HoMCLT strains under different carbon sources and stress conditions. The growth diameter of the mutant strains on plate medium was measured. There are two separates independent transformants for each mutant. Values represent the means ±*SE* of three independent samples. There was no significant difference between data at $p > 0.05$ based on unpaired Student's *t* tests.
